# Comparison of marginal gap in implant-supported hybrid nanoceramic crowns fabricated by additive vs. subtractive manufacturing

**DOI:** 10.1186/s12903-026-07710-2

**Published:** 2026-02-02

**Authors:** Shaimaa Omar, Enas A. Elshenawy, Sherif M. Elsharkawy

**Affiliations:** 1 Fixed Prosthodontics department, Faculty of Dentistry, Tanta University, Tanta, 31773 Egypt; 2 Dental Biomaterials department, Faculty of Dentistry, Tanta University, Tanta, 31773 Egypt

**Keywords:** Additive manufacturing, Computer aided design, Computer aided manufacturing, Aging, Dental prosthesis, Dental materials

## Abstract

**Background:**

Achieving optimal marginal accuracy is a critical factor in the long-term success of implant-supported crowns. The purpose of this study was to evaluate and compare the marginal gap of implant-supported novel hybrid nanoceramic crowns, Nanoksa G-Plus and Nanoksa BioGuard, which are innovative due to their unique composition when manufactured using two distinct digital workflows—milling and 3D printing—to determine which technique provides superior precision for this new class of restorative materials.

**Methods:**

Twenty implant fixtures were embedded into epoxy resin blocks. Stock straight titanium abutment was screwed to each fixture according to manufacturer recommendations. The samples were divided into 2 groups according to the technique of fabrication of Nanoksa hybrid nanoceramic crowns (*n* = 10/group). Group I subtractive technique using Nanoksa G Plus discs and group II additive technique using Nanoksa BioGuard. Stock abutments were scanned using a laboratory scanner, the crowns were fabricated and cemented with resin cement after pretreatment of all crowns according to manufacturer recommendation. Using a stereomicroscope (35X), the marginal gaps were assessed both prior to cementation and following thermomechanical accelerated aging. Mean values were calculated, and differences between groups were tested for statistical significance using paired t-test and Student t-test. A value of (*P* < 0.05) was considered statistically significant.

**Results:**

Significant differences were detected in the marginal gaps between the groups. Before cementation and following thermomechanical accelerated aging, 3D printing crowns showed a statistically significant larger marginal gap distance (59.71 and 63.83 μm, respectively) than the milled one (34.08 and 37.76 μm).

**Conclusion:**

The milling technique demonstrated significantly lower discrepancy values compared to additive technique, indicating superior marginal fit for Nanoksa hybrid nanoceramic crowns. Clinically, these findings suggest that utilizing milling for these restorations may enhance long-term outcomes by reducing the risks associated with larger gaps.

## Background

The success of implant restorations relies not only on successful osseointegration but also significantly on the success of the implant superstructure [1]. An implant-supported prosthesis’s clinical success is influenced by the restorative material [2]. The use of restorative materials with various mechanical and chemical qualities, like resin matrix ceramic, has been made easier by developments in computer-aided design and computer-aided manufacturing (CAD-CAM) [3]. Its unique composition, which combines ceramic components with polymer matrix, offers a number of advantages for implant-supported restorations. By improving shock absorption and force distribution, these materials help reduce the amount of stress applied to the surrounding bone of the implant. Their endurance is increased by their lower elasticity modulus as compared to traditional ceramics, which is crucial for the long-term success of implant-supported restorations [4]. Additionally, their superior edge fitting and ease of machining increase their ability to improve the biomechanical functionality of prosthetic restorations [5].

Nanoksa G-Plus, a hybrid nano-ceramic material containing nano-carbon and nano-zirconia, is presented by its manufacturer as a comprehensive system for definitive dental implant restorations. The material is indicated for creating durable crowns, bridges, and full-arch dentures, with the carbon element being a key factor in its extended longevity [6]. According to the manufacturer, the material exhibits minimal water sorption, approximately 0.1% after 24 h of immersion in water at 23 °C [6]. Owing to its highly crystalline structure, Nanoksa G-Plus demonstrates insolubility in conventional solvents at room temperature. Based on standardized mechanical testing, the reported yield strength ranges between 90 and 100 MPa, while tensile strength at fracture is approximately 95–110 MPa [7].

The special blend used in the formulation of Nanoksa G-Plus is designed to offer both powerful resistance to wear and breakage and smooth cutting. Its exceptional strength, longevity, shock absorption, and monomer-free, biocompatible nature are all highlighted by the manufacturer. With high transparency and a color that mimics natural teeth, the material ensures high aesthetics, coupled with excellent color stability and abrasion resistance [6]. Additionally, they do not require firing and they are not brittle [8, 9].

Nanoksa BioGuard is a novel 3D-printing hybrid nanoceramic material developed for fabrication of permanent fixed dental restoration and long bridge dental implant restorations. It comprises a hybrid resin matrix reinforced with nano-zirconia, nano-ceramic and nanocarbon fillers which aim to enhance materials’ mechanical performance and optical properties. The incorporation of antibacterial nano compounds is intended to reduce bacterial colonization on the restoration surface, contributing to improve oral and longevity. Nanoksa BioGuard is compatible with SLA and DLP 3D printing systems, facilitating chair side fabrication of definitive restoration in single clinical visit [10].

CAD/CAM systems are now broadly divided into subtractive manufacturing (SM) and additive manufacturing (AM). Compared to conventional dental laboratory techniques, SM provides significant benefits, such as the incorporation of novel materials, reduced labor and superior quality control [8, 11]. However, the technology is limited by a number of challenges, such as significant material waste from unused blocks, the need to replace milling tools after predetermined usage cycles, limited capacity for surface morphology replication because of machine axis and milling instrument size limitations, and the possibility of microscopic crack formation during the milling of ceramic materials [12, 13].

Additive manufacturing (3D printing) of permanent fixed dental prosthetics is a growing trend in dentistry [14]. This technology enables the fabrication of complex geometries, including undercuts and regions that are inaccessible to conventional milling techniques, while minimizing the need for tool replacement and maintenance. In addition, additively manufactured components may exhibit lower residual stresses, which can be advantageous for the structural integrity of complex restorations [15–17].

Optimal marginal adaptation is essential for minimizing cement thickness and avoiding microleakage, which can compromise the restoration [18, 19]. The implant, supporting tissues, and supra-structure may suffer from an inadequate fit [20]. A wide gap at the abutment level results in faster cement dissolution, creating recesses for plaque accumulation and bacterial adherence which leads to bone resorption around implants [21]. Furthermore, a thicker cement layer will increase interfacial stresses and polymerization shrinkage, which could lower the restorations’ resistance to fracture. Consequently, adequate fit between implant components is crucial to minimize mechanical and biological complications [22].

The appropriate marginal gap width has not been agreed upon. According to McLean and Von Fraunhofer’s study [23], a marginal gap up to 120 µ is clinically appropriate.

Replicating the challenging conditions during service via artificial aging is an approach designed to predict the materials’ endurance in the oral cavity [24].

Donmez et al. [22] evaluated the marginal adaptation of implant-supported crowns by comparing crowns fabricated from 3D-printed resin composite to three milled crown materials, namely reinforced composite, polymer-infiltrated ceramic, and hybrid ceramic and found 3D-printed to have the least marginal gap. Sayed et al. [25] compared the marginal integrity of 3D printed hybrid resin-ceramic crowns to that of milled lithium disilicate and milled hybrid resin ceramic crowns and found the marginal gap values to be the same across all the groups.

Due to the limited available evidence on the marginal accuracy of implant-supported hybrid nanoceramic crowns (Nanoksa G Plus and Nanoksa BioGuard) fabricated on stock straight titanium abutments using different manufacturing techniques, the present study was undertaken to evaluate and compare the marginal gap of implant supported hybrid nanoceramic crowns using two different techniques of fabrication. The null hypothesis for this study was that no statistically significant difference in marginal accuracy exists between implant-supported novel hybrid nanoceramic crowns using subtractive and additive manufacturing (AM) techniques.

## Methods

**Fig. 1:**
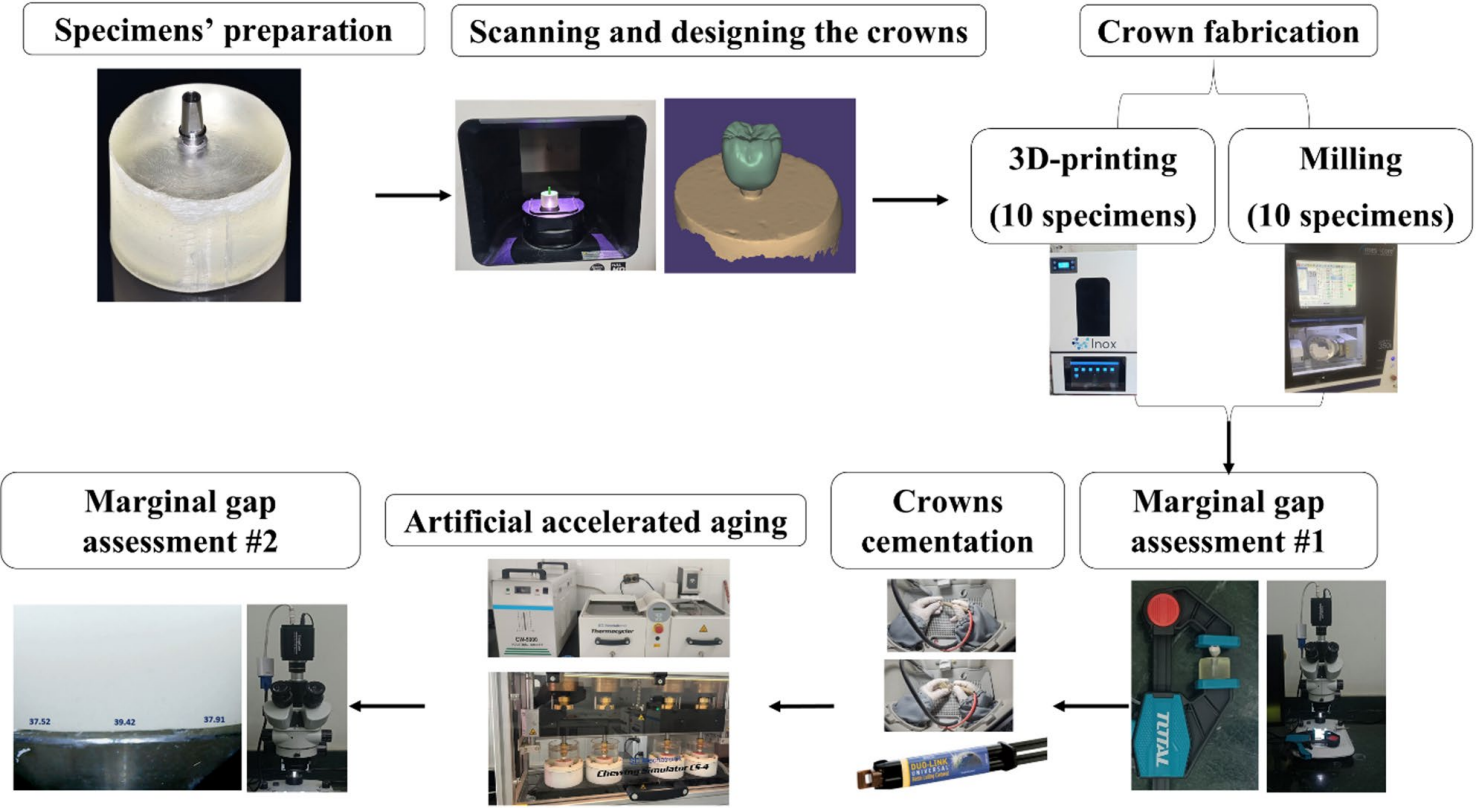
Flow chart of the study design

The current study was carried out as a controlled experimental study Fig. 1.

A sample size calculation was executed based on outcomes of a previous study [26] employing a statistical software program (G*Power 3.1.9.2; Kiel University). Utilizing an alpha level of significance of 0.05 and a desired power of 80%, the analysis determined that 10 specimens/group were required.

The materials used in the study are listed in Table 1.

**Table 1:** Materials used in the study

Material	Brand name	Composition	Manufacturer
Dental implant	Implance Dental Implant	Commercially pure titanium grade4	Implance, Istanbul, Turkey
Polymer-infiltrated nanoceramics CAD-CAM disc	Nanoksa G Plus	High performance polymers filled with Nano-Zirconia and Carbon	Inox, Sheridan, USA
Polymer-infiltrated nanoceramics CAD-CAM 3D printed resin	Nanoksa BioGuard	Methacrylates, Nano-Ceramics, High-Concentration Nano-Zirconia, Nanocarbon Enhancement, Anti-Bacterial Nano Compound, Photo-initiator, Inhibitor, Pigment	Inox, Sheridan, USA
Resin cement	DUO- LINK	**Catalyst**: Glass fiber, 10-methacryloyloxydecyl dihydrogen phosphate, silica amorphous. **Base**: Calcium base filler, Glass filler, Bisphenol A di glycidyl methacrylate, di methacrylates, 2-hydroxyethyl methacrylate, ytterbium fluoride, initiator, amorphous silica.	Bisco, Inc., Schaumburg, IL, USA
Ceramic primer	Z primer	Combination of two active monomers, MDP, a phosphate monomer, and BPDM, a carboxylate monomer.	Bisco, Inc., Schaumburg, IL, USA
Silane coupling agent	Porcelain primer	HEMA, Ethanol, purified water, silane coupling agent, methacrylate ester monomer HEMA (2-hydroxyethyl methacrylate)	Bisco, Inc., Schaumburg, IL, USA

### Specimens’ preparation

Twenty dental implant fixtures (Implance Dental Implant, Istanbul, Turkey) with diameter 4.8 mm and length 10 mm were fixed centrally in custom made metallic mold using sticky wax (Kerr Corporation, California, USA). The long axis positioning of the implant fixture was standardized using a surveyor (Ney surveyor; Dentsply Sirona, North Carolina, USA). Epoxy resin was used to fill the mold as its elastic modulus (approximately 16.8 GPa) closely simulates that of human bone [27]. The base and hardener were manually spatulated following manufacturer recommendations. The mix then was poured into a metallic mold within fixture in its place. After setting, epoxy models were removed from the metallic mold and checked for accuracy.

Stock straight titanium abutment (7 mm height, 2 mm collar height) was screwed to each implant fixture according to manufacturer instructions using torque driver at a torque level of 25 Ncm. The specimens were divided into 2 groups (*n* = 10 each) according to the fabrication techniques, group I samples were milled from hybrid nanoceramics (Nanoksa G-plus ^®^, Ionx, USA) while the group II samples were 3D printed with hybrid nanoceramics resin (Nanoksa BioGuard ^®^, Ionx, USA).

### Scanning and designing the crowns

The screw access hole of the abutment was sealed, each abutment was painted with anti-reflective paint (SCAN-LAC, 3D LAC, Germany) and allowed 5 s to dry. All abutments across all experimental groups were treated using the same paint and application protocol. Therefore, any potential dimensional influence was systematic and did not affect intergroup comparisons. All aspects were scanned with extraoral scanner (DOF Freedom HD, Seoul, Korea). A three-dimensional model was created, and information was saved as Standard Tessellation Language file (STL).

**Fig. 2:**
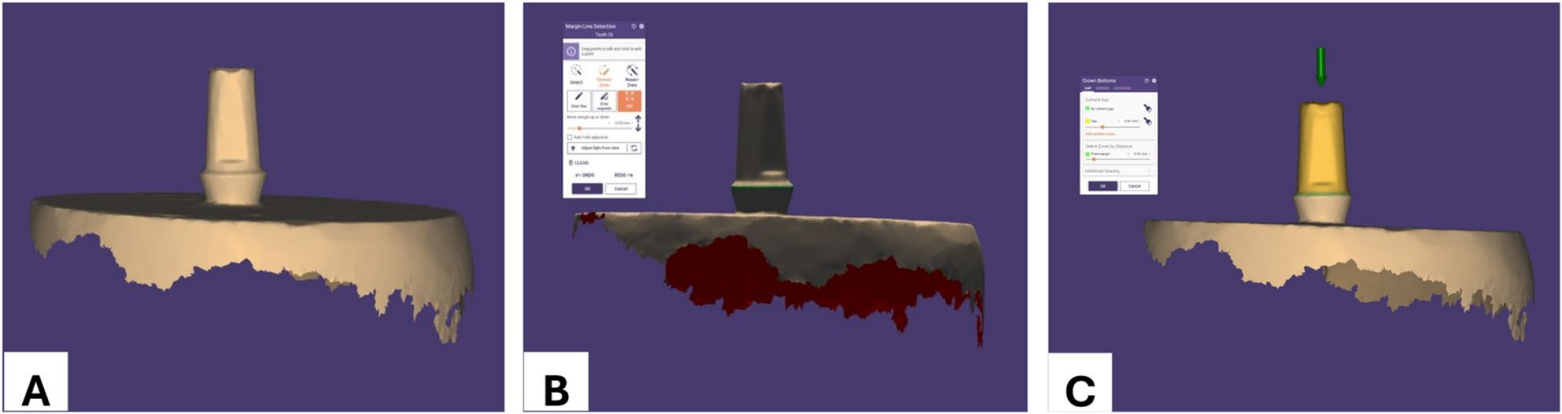
Designing steps. **A** 3D-model of the scanned abutment surface; **B** Detection of the margin; **C** Cement gap determination

Designing of the crowns was done using ExoCad^®^ software (ExoCad 2020 GmbH, Darmstadt, Germany). The margin was drawn on the 3D virtual model as a closed green line to detect the marginal line by series of clicking around margin of the abutment. Cement space was set to be 60 μm circumferentially leaving 1 mm above the margin Fig. 2.

The molar design was adjusted as follows; 10 mm crown length from buccal cusp to implant platform, 10 mm width mesio-distal; and 10 mm bucco-lingual. The occlusal surface was shaped with two flat sides converging to midline; thus, the central sulcus was located exactly in the center of the plane perpendicular to the long axis of the crown.

### Crown fabrication

Nanoksa G Plus disc was fixed in the spindle of the milling machine (i mes- i core, Gmbh, Germany) to start the milling all specimens employing the same previous design to achieve standardization and uniformity of both crown size and design across the group.

For the 3D printing group, design file was converted and saved in STL format to be compatible with the 3D printer (Inox S2, USA). Prior to importing the STL file, the printing platform and the Nanoksa BioGuard resin material were thoroughly prepared. Upon successful importation, the printing parameters were configured, and the fabrication process was initiated. Following printing, the samples were cleaned with 96% ethanol and post-cured for five minutes in a UV light cure box that worked in a vacuum with nitrogen gas (Inox, USA). The complete seating of all crowns within the two groups was confirmed by examining them on their abutments using magnifying loupes (Univet Loupes Spa, Italy).

### Marginal gap assessment

**Fig. 3:**
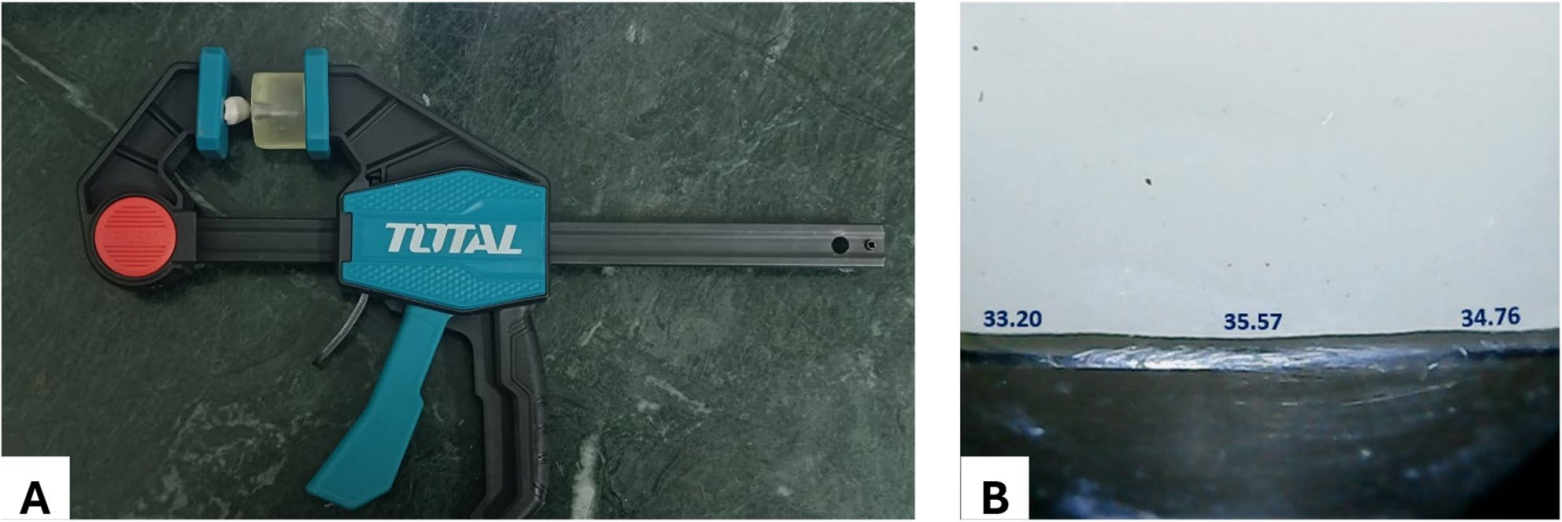
Marginal gap assessment. **A** stabilizing the specimen, **B** stereomicroscope measurement before cementation

Before cementation, the crowns’ vertical marginal gap was measured to evaluate their fit accuracy. The measurement process involved stabilizing the crown with a holding device, then examining it under magnification using a stereomicroscope at 35X (Zeiss, Germany). Fig. 3. All stereomicroscopic measurements were performed by a single trained examiner to eliminate inter-examiner variability. The examiner was blinded to group allocation, and specimens were coded prior to measurement to minimize potential measurement bias.

After that, the gap was captured by camera and examined using specialized image analysis software (Olympus DP2-SAL; Olympus Corp, Japan) Fig. 3. Six reference points were set up on each crown to assess the marginal gap values: mesiobuccal, midbuccal, and distobuccal on the buccal side, and mesiolingual, midlingual, and distolingual on the lingual side. All six points were consistently marked on the epoxy resin using a permanent marker for standardization the measurement points for all specimens. These six measurements were averaged to obtain a single mean marginal gap value per crown, and the per-crown mean was used for statistical analysis.

### Crowns cementation

Each abutment and the fitting surfaces of crowns were air-born particle abraded (Air-born particle-abrasive device, Renfert, Germany) using 50 μm Al_2_O_3_ particles at 1.5 bar according to manufacturer instructions. Ceramic primer (Z prime, Bisco, Inc., Schaumburg, IL, USA) was applied to the treated surface of the abutment using micro-brush (Unipack, Unipack medical Corp, China), allowed to react for 60 s, and dried with sufficient air syringe. Silane coupling agent (Porcelain primer, Bisco, Inc., Schaumburg, IL, USA) was then applied with a micro-brush for 30 s according to the manufacturer’s instruction and dried with sufficient air syringe.

Strictly adhering to the manufacturer’s recommendations, all crowns were cemented using Duo-Link adhesive resin cement. The crowns were gently seated on the corresponding abutments, initially with finger pressure and then by applying a controlled load of 5-kg static load (49 N) for 10 min in order to guarantee uniformity and full seating until set [28, 29]. The same seating procedure was applied for all specimens and experimental groups to ensure consistency and minimize variability related to seating force and ensured valid intergroup comparisons.

Excess cement at margin was light cured for 2 s using LED curing unit (Woodpecker, LED, Germany) and removed with an explorer (MA Dental, Dentaltix, Spain). Light cured the margin for 20 s/surface to complete cement curing.

### Artificial accelerated aging

The crowns within two groups were first subjected to a thermal aging protocol consisting of 5,000 cycles using an automated thermal cycler (SD Mechatronik thermocycler CW-5000, GmbH, Germany). This process involved alternating between a low temperature of 5 °C and a high of 55 °C (25 s dwell time-10 s lag time). This thermal cycling is considered clinically equivalent to six months of use and is commonly used to predict material performance under oral conditions [30, 31]. Following this, all samples underwent mechanical cyclic loading using a chewing simulator (SD Mechatronik GmbH, Germany). This simulation involved 75,000 cycles (50 N load- 1–1.6 Hz- wet conditions), which also approximates six months of clinical function [32].

### Marginal gap after aging

Upon completion of aging protocol, the marginal gaps were measured a second time following the previously mentioned steps.

### Statistical analysis

Collected data was statistically analyzed using paired T-test to compare between marginal gap in the same group before and after aging, and unpaired T-test between the two groups at each stage. Statistical Package for Social Science (SPSS version 25, SPSS Inc, IBM) was used. The value of (*P* < 0.05) indicated significance.

## Results

The means and standard deviations for marginal gap measured in micrometer (µm) and T-test results are presented in Table 2. Regarding comparison between the two techniques, milled group showed statistically significant lower marginal gap values compared to 3D-printed group both before cementation and after aging (*P* < 0.001). The marginal gap increased significantly following thermomechanical accelerated aging compared to before cementation, regardless of the production method (*P* < 0.001).

**Table 2:** Marginal gap results (µm) (mean±SD) and T-test results

	Marginal gap		Group		T-Test	
			**Milled group**	**3d Printed group**	**T**	***P*** **-value**
	Before cementation	Range	32.98 - 35.36	58.49 - 60.51	69.50	<0.001*
		Mean ±SD	34.08 ± 0.85	59.71 ± 0.79		
	After aging	Range	36.99 - 38.88	62.54 - 64.56	94.81	<0.001*
		Mean ±SD	37.76 ± 0.64	63.83 ± 0.57		
Differences		Mean ±SD	3.67 ± 0.86	4.12 ± 1.08		
Paired T-Test	T		13.48	12.00		
	*P* -value		<0.001*	<0.001*		

*Significant at *P *≤ 0.05

## Discussion

In this study, marginal accuracy of implant supported hybrid nanoceramic crowns was evaluated by measuring the vertical marginal gap at six standardized locations per crown and then comparing subtractive and additive techniques for novel hybrid ceramic materials to determine which technique provides superior precision. The null hypothesis was rejected since there was a substantial difference in the marginal accuracy between the two groups.

The implant fixture was centrally positioned within a metallic mold using a dental surveyor to ensure accurate parallel alignment [8].

Stock straight titanium abutments were selected because of their wide availability, ease of handling, and ability to ensure specimen standardization across all groups [33]. The abutments were secured to the implant fixtures following the manufacturer’s instructions using a torque of 25 Ncm, generating a compressive force that maintains intimate contact between the implant and abutment interfaces [34]. Abutments were scanned using a desktop scanner commonly employed in dental laboratories to obtain high-precision three-dimensional data for digital restoration design. This scanning system integrates advanced light and laser technologies with a rotating platform, thereby minimizing external interferences and enhancing scan quality [35, 36]. Compared with conventional impression techniques, the digital impression approach offers superior accuracy, improved time efficiency, procedural simplicity, and greater patient comfort [37]. Since metallic abutments are highly reflective and may compromise scanning accuracy, a scanning spray was applied to reduce optical reflections and prevent surface highlights that could interfere with accurate data acquisition [28]. To ensure precise marginal adaptation of the restorations, the cement spacer was standardized at 60 μm, beginning 1 mm above the crown margin [28, 38].

To simulate clinical scenario, resin cement was used in the cementation of the crowns and a screw access channel was made to achieve optimal luting and successful clearing of residual cement [39].

Considerable advancements in CAD/CAM technology have made it easier to integrate new materials with a variety of properties and simplified a number of laboratory steps [40, 41]. Hybrid nanoceramic was chosen to be used in the current study as it represents an appealing alternative for aesthetic situations beside their resilience, improved force distribution, machinability and marginal fit [42, 43]. While resin-based prostheses have originally been created via SM [44], printable hybrid nano-ceramics have just entered the market, making AM a feasible choice for permanent restorations [45]. As a result, the purpose of this study was to assess and compare the effectiveness of the additive and subtractive methods.

The marginal gap was evaluated via a non-invasive, accurate, and reproducible direct observation technique using an external measurement microscope set at 35X magnification [46]. This method can calculate the marginal gap throughout the whole sample, and for standardization, six consistent measurement locations were marked on an epoxy resin base using an indelible marker [47]. Following these marked locations, six measurements were recorded for each crown both before and after cementation [48]. The number of measurements taken was deemed adequate to yield a consistent and reliable estimate for the gap size, thereby permitting an accurate evaluation of the restoration’s circumferential fit [49].

Due to the expense and duration of clinical trials laboratory-based accelerated aging specifically thermal cycling, is used to predict the lifespan of ceramic restorations by replicating in vivo conditions. This study conducted 5,000 cycles, simulating about six months of clinical aging [30]. Calibration of the masticatory simulation parameters was performed according to the relevant physiological values documented in published literature [32]. In order to simulate roughly six months of regular clinical service, mechanical cycling up to 75,000 cycles was used in our study [32].

In clinical terms, the highest acceptable marginal gap for implant-supported restorations is still widely debated, with documented acceptable values in the literature varying substantially from 50 to 200 μm [50]. A significant study by McLean and von Fraunhofer [23] concluded that a marginal gap of less than 120 μm is clinically acceptable. A stereomicroscope was used to assess the marginal gap of the restorations, as it offers a combination of high gap detection accuracy, ease of use, and low cost [51]. To accurately assess the primary precision of each crown restoration and control for potential confounding factors, the marginal gap was measured prior to cementation. This methodology, supported by other authors, deliberately eliminates variables such as cement film thickness, viscosity, and mixing technique [52, 53].

The average marginal gap for all hybrid nanoceramic crowns, regardless of the manufacturing technique, was less than 64 μm signifying that every specimen met acceptance. A direct comparison with another study was not possible due to the small number of studies examining the marginal fit of Nanoksa G plus and Nanoksa BioGuard crowns. These findings, however, could be compared with research by Soliman et al. [54] that examined the fit of crowns made using various CAD/CAM materials. They found that hybrid ceramic (Vita Enamic) resulted in smoother margins during milling of crowns which gives better marginal adaptation. These results in agreement with previous studies who related the higher marginal accuracy of hybrid ceramic, compared to the tested ceramics, to the omission of additional post-milling fabrication [12, 55]. The results are also in agreement with other studies who concluded that milled hybrid materials exhibit improved adaptation due to their low brittleness nature when compared to ceramic based materials [19, 56]. In agreement to the present study, Sayed et al. [25] reported lower marginal gap values for milled Nanoksa G-plus crowns compared with 3D-printed Nanoksa.

Bioguard crowns; however, this difference did not reach statistical significance.

Conversely, Suksuphan et al. [17] reported lower marginal gap values for 3D-printed polymeric crowns compared with milled polymeric and polymer-infiltrated glass ceramic crowns. Similarly, Donmez et al. [22] evaluated the marginal fit of implant-supported crowns and found that 3D-printed resin composite crowns demonstrated the smallest marginal gaps when compared with milled crowns fabricated from reinforced composite, polymer-infiltrated ceramic, and hybrid ceramic materials. The discrepancies between these findings and the present study may be attributed to differences in the chemical composition of the evaluated resin composite materials, including variations in resin matrix formulation and filler type or content.

Layer-by-layer 3D printing can lead to errors because of attributes like surface imperfections and layer thickness, which is an adjustable parameter that should be optimized to achieve optimal restoration properties and performance [57, 58]. Furthermore, the more extensive post-production procedures required for additive manufactured hybrid nanoceramic restorations, compared to milled ones, may introduce additional disparity in the marginal fit [59]. The removal of the printing support structures that were attached during the fabrication process may have also contributed to the greater marginal gap in 3D-printed group [60]. The overall dimensional accuracy may be adversely affected by warping or insufficient material hardening resulting from either overcuring or undercuring [61].

After cementation, each group exhibited a higher mean marginal gap compared to the pre-cementation values. The milled Nanoksa G plus crowns (Group M) exhibited mean gaps of 34.08 μm before and 37.76 μm after cementation and aging, while the 3D printed crowns (Group P) recorded mean gaps of 59.71 μm and 63.83 μm, correspondingly. Many studies comparing marginal gap before and after cementation have found that the use of cement negatively impacts the fit, causing a consistent increase in the marginal gap following the procedure as even a minimal amount of cement contributes an extra layer of material [59, 62]. The statistically significant increase in marginal gap observed after thermocycling is consistent with the findings of Sayed et al. [25], who attributed this effect to the degradative impact of thermocycling on resin luting cement.

### Limitations

The current study’s limitation is the lack of an internal fit assessment. Improved adaptation of the intaglio surface enhances complete seating of the crown, thereby contributing to its clinical success and long-term longevity. Conversely, irregularities on the intaglio surface may hinder proper seating, potentially leading to marginal discrepancies and the formation of open margins [63]. Therefore, this area warrants further investigation. In addition, longer aging periods may be beneficial in capturing the long-term effects on restorations.

Because of use of a laboratory scanner, the results might not capture the clinical scenario. In the clinical environment, the accuracy of CAD/CAM crowns is often adversely affected by factors like gingival bleeding, moisture, and limited mouth opening during intraoral scanning.

## Conclusions

Within the limitations of this in vitro study, the findings highlight the influence of manufacturing approach and cementation on the marginal adaptation of implant-supported crowns. The observed differences between additive and subtractive techniques, together with the effect of cementation, underline the need for careful material selection and standardized luting procedures when evaluating marginal accuracy. Further well-designed clinical studies are required to determine their relevance under clinical conditions.

## Data Availability

All data used and analyzed during the current study are available from the corresponding author on reasonable request.
